# Supplementary material for: Fine-mapping of qTGW2, a quantitative trait locus for grain weight in rice (Oryza sativa L.)
Source: PeerJ. 2020 Mar 4;8:e8679. doi: 10.7717/peerj.8679 (PMC7060756; doi:10.7717/peerj.8679)
Supplement: Table S1 [file peerj-08-8679-s001.docx]

**Table S1 The markers used in this study**

| **Name** | **Type** | **Chr** | **Primer** |  | **AT (^o^C)** | **Gel** | **Enzyme** |
| --- | --- | --- | --- | --- | --- | --- | --- |
|  |  |  | **Forward (5'-3')** | **Reverse (5'-3')** |  |  |  |
| Wn22892 | InDel | 1 | CACACTCGTCCTTATCCAGA | TCTTTCAGGAGAGATAGAGCA | 52 | 6% PAGE |  |
| Wn30334 | CAPS | 1 | GCATAGGTATATACACGTCCTT | TTTACCATGATGAGACGTGTAGG | 50 | 2% agarose | *Taq*I |
| Wn34352 | InDel | 1 | TTTTCCAGAGACATCCGATA | GTTGCCCTAGATTACCCTC | 52 | 6% PAGE |  |
| Tw31911 | InDel | 2 | AGAGCTATGCTGGCTTCGAT | CTTAGGTACACGGCTCGAGA | 55 | 6% PAGE |  |
| Tw32437 | InDel | 2 | TCTACTTACAAACCGAACTCGAT | GAGCTCGCTTGCCTATGCTT | 55 | 6% PAGE |  |
| Tw35293 | InDel | 2 | TCACGTTGACATAAGTGG | ATTATTTGTCACTGATAGGT | 50 | 6% PAGE |  |
| Tw35277 | InDel | 2 | CGTAAGCCAGAAAGTCTTCA | ATTGAGCCACATCACCTA | 55 | 6% PAGE |  |
| Tw35395 | InDel | 2 | AAAATGACCTGATCCCAA | GGCAAGCATGTTAACTCTG | 55 | 6% PAGE |  |
| Tv963 | InDel | 12 | AGATTGCTCTAAGTAACAAG | AACTAGTGCAAGATAGTGTTC | 55 | 6% PAGE |  |

**Notes.**

Chr, chromosome number; AT, annealing temperature.
