# Supplementary material for: Fine-mapping of qTGW2, a quantitative trait locus for grain weight in rice (Oryza sativa L.)
Source: PeerJ. 2020 Mar 4;8:e8679. doi: 10.7717/peerj.8679 (PMC7060756; doi:10.7717/peerj.8679)
Supplement: Table S2 [file peerj-08-8679-s002.docx]

**Table S2 Annotated genes in the 103-kb region for *qTGW2***

| Gene_name | Description |
| --- | --- |
| LOC_Os02g57630 | ubiquitin carboxyl-terminal hydrolase, family 1, putative, expressed |
| LOC_Os02g57640 | KH domain containing protein, putative, expressed |
| LOC_Os02g57650 | no apical meristem protein, putative, expressed |
| LOC_Os02g57660 | phosphatidylinositol-4-phosphate 5-kinase, putative, expressed |
| LOC_Os02g57670 | ribosomal L9, putative, expressed |
| LOC_Os02g57690 | kelch repeat protein, putative, expressed |
| LOC_Os02g57700 | protein kinase, putative, expressed |
| LOC_Os02g57710 | signal peptide peptidase-like 2B, putative, expressed |
| LOC_Os02g57720 | aquaporin protein, putative, expressed |
| LOC_Os02g57730 | hypothetical protein |
| LOC_Os02g57740 | expressed protein |
| LOC_Os02g57750 | protein binding protein, putative, expressed |
| LOC_Os02g57760 | O-methyltransferase, putative, expressed |
| LOC_Os02g57770 | glycosyl hydrolases family 16, putative, expressed |
| LOC_Os02g57780 | expressed protein |
| LOC_Os02g57790 | ZOS2-19 - C2H2 zinc finger protein, expressed |
